# Heart Rate Variability Biofeedback to Treat Anxiety in Young People With Autism Spectrum Disorder: Findings From a Home-Based Pilot Study

**DOI:** 10.2196/37994

**Published:** 2022-08-26

**Authors:** Helen Coulter, Mark Donnelly, John Mallett, W George Kernohan

**Affiliations:** 1 South Eastern Health and Social Care Trust County Down United Kingdom; 2 Ulster University Newtownabbey United Kingdom; 3 Ulster University Coleraine United Kingdom

**Keywords:** autism, anxiety, biofeedback, remote intervention, mobile phone

## Abstract

**Background:**

People with autism spectrum disorder (ASD) frequently experience high levels of anxiety. Despite this, many clinical settings do not provide specialist ASD mental health services, and demand for professional support frequently outstrips supply. Across many sectors of health, investigators have explored digital health solutions to mitigate demand and extend the reach of professional practice beyond traditional clinical settings.

**Objective:**

This critical appraisal and pilot feasibility study examines *heart rate variability* (HRV) *biofeedback* as an approach to help young people with ASD to manage anxiety symptoms outside of formal settings. The aim is to explore the use of portable biofeedback devices to manage anxiety, while also highlighting the risks and benefits of this approach with this population.

**Methods:**

We assessed the feasibility of using home-based HRV biofeedback for self-management of anxiety in young people with ASD. We adopted coproduction, involving people with ASD, to facilitate development of the study design. Next, a separate pilot with 20 participants with ASD (n=16, 80% male participants and n=4, 20% female participants, aged 13-24 years; IQ>70) assessed adoption and acceptability of HRV biofeedback devices for home use over a 12-week period. Data were collected from both carers and participants through questionnaires and interviews; participants also provided single-lead electrocardiogram recordings as well as daily reports through smartphone on adoption and use of their device.

**Results:**

Pre-post participant questionnaires indicated a significant reduction in anxiety in children (*t*_6_=2.55; *P*=.04; Cohen *d*=0.99) as well as adults (*t*_7_=3.95; *P*=.006; Cohen *d*=0.54). Participant age was significantly negatively correlated with all HRV variables at baseline, namely high-frequency heart rate variability (HF-HRV: *P*=.02), the root mean square of successive differences in normal heartbeat contractions (RMSSD: *P*=.02) and the variability of normal-to-normal interbeat intervals (SDNN: *P*=.04). At follow-up, only SDNN was significantly negatively correlated with age (*P*=.05). Levels of ASD symptoms were positively correlated with heart rate both before (*P*=.04) and after the intervention (*P*=.01). The majority (311/474, 65.6%) of reports from participants indicated that the devices helped when used. Difficulties with the use of some devices and problems with home testing of HRV were noted. These initial findings are discussed within the context of the strengths and challenges of remotely delivering a biofeedback intervention for people with ASD.

**Conclusions:**

HRV biofeedback devices have shown promise in this pilot study. There is now a need for larger evaluation of biofeedback to determine which delivery methods achieve the greatest effect for people with ASD.

**Trial Registration:**

ClinicalTrials.gov NCT04955093; https://clinicaltrials.gov/ct2/show/NCT04955093

## Introduction

### Background

Autism is a lifelong developmental disability that affects how people communicate and interact with the world [1]. Substantial changes to how autism is understood and defined have taken place over the years since the term was initially described [2]. Autism is currently viewed as a neurodevelopmental disorder that develops in childhood, and the terms *Kanner autism*, *Asperger disorder*, *childhood disintegrative disorder*, and *pervasive developmental disorder not otherwise specified* have been replaced with the collective term *autism spectrum disorder* (ASD) [3]. All participants in this study had been diagnosed with ASD in specialist health service assessments clinics using standardized measures. None of the participants were diagnosed with a learning disability, and all had attended mainstream education. Although the term Asperger syndrome is still used by both clinicians and some able people with autism, the term *ASD* will be used throughout this paper to describe the condition.

Approximately 1% of the British population are likely to have some form of ASD, with studies reflecting this prevalence both in children [4] and adults [5]. Epidemiological studies have shown a dramatic increase in the numbers of people being diagnosed with ASD. In the United States, the Centers for Disease Control and Prevention reported a prevalence rate of ASD in children of 1 in 88 [6], which was updated to 1 in 59 in 2018. The reasons for these apparent increases have been debated but may be related to changing definitions of the disorder, increased awareness of the condition in women, and recognition of the condition in people with no learning disability [7].

People with ASD can experience a range of mental health difficulties [8,9] and present with high levels of anxiety [10]. A range of interventions exist to treat anxiety in ASD [11]. There is some evidence for effectiveness of nonpharmacological interventions such as cognitive behavioral therapy adapted for people with ASD [12], but availability of such interventions is limited by poor adoption rates [13] and scarcity of mental health services for people with ASD [14].

There is evidence that digital health interventions can aid compliance with traditional treatments and help to reduce increasing demands on health provision, while extending the reach of professional practice beyond traditional clinical settings [15].

### Digital Health Interventions

Digital solutions can be tailored to the needs of people with ASD and can be associated with less stress than that reported in face-to-face interventions [16]. Technology may be used both to deliver interventions and augment communication and social interaction [17]. Emerging digital health interventions are being investigated to help those with ASD to understand and control their reactions [18] and to help them better interpret social situations [19]. A type of intervention that has been found to reduce symptoms of anxiety in a range of populations makes use of *biofeedback* [20,21], which has been defined as “a self-regulation technique through which patients learn to voluntarily control what were once thought to be involuntary body processes” [22]. Biofeedback actively involves the user, enabling them to change certain physiological responses to improve health [23].

### Biofeedback

A variety of biofeedback equipment now exists for both psychophysiological stress profile assessment [24] and for biofeedback training [23], typically categorized into two types: large professional systems and small portable devices intended for personal use. Treatment protocols exist for a wide range of physical and mental health conditions, using either a single sensor or multiple sensors with evaluations of efficacy for each condition [25].

Although biofeedback has been used since the 1970s, there is now growing interest in its use in stress management [26]. Several systematic reviews highlight evidence supporting biofeedback as a cost-effective intervention to help manage anxiety in clinical populations [21,27]. A review of the types of biofeedback modalities and devices currently being evaluated for stress management has been carried out by Yu et al [28]. Many devices have only been tested in nonclinical populations or in laboratory environments, and the importance of testing devices in real-world situations with diverse types of clinical populations has been noted [29].

To date, the most widespread use of biofeedback in individuals with ASD has involved the use of neurofeedback [30,31]. Recent studies show some positive effects in areas such as attention and social skills; however, systematic reviews investigating neurofeedback in ASD have not shown significant results in reducing symptoms such as anxiety [32,33]. An alternative type of biofeedback that has been used to manage anxiety has been termed *resonant frequency feedback*, more commonly known as *heart rate variability* (HRV) biofeedback [34].

### HRV Biofeedback

HRV was first recognized 60 years ago as an indicator of fetal distress [35] and then found to be affected by breathing frequency [36]. HRV reflects the activity of both the sympathetic and parasympathetic activities of the autonomic nervous system and is reported in both time and frequency domains [37]. A review of HRV metrics has been produced [38], with population norms identified [39]. HRV is affected by age [40], the environment, breathing rate, and blood pressure, and it is subject to regulation of the autonomic nervous system [41]. HRV is frequently used as a physiological marker in stress detection [42]. It is also an index of adaptability and of the ability to self-regulate behavior [43].

A number of theoretical models have been proposed describing the links between HRV and health, mediated through connections between the heart and the brain [44,45]. The polyvagal theory suggests that impairments in the autonomic nervous system involving vagus nerve regulation, termed the *social engagement system,* are features of several disorders, including ASD.

HRV has been used in conjunction with sensor technology to develop *HRV biofeedback* [46,47]. Training in HRV biofeedback involves *resonant frequency breathing*, which has been associated with *respiratory sinus arrhythmia* where heart rate acceleration and deceleration rates synchronize with respiration and occur when breathing is slowed to a rate between 4.5 and 7.0 breaths per minute [41]. To support the maintenance and development of resonant frequency breathing, individuals can practice paced breathing or use portable *home trainer* biofeedback devices.

Portable biofeedback devices typically derive HRV using infrared photoplethysmography (PPG), which measures blood flow, usually through either the fingertip or the earlobe. Peripheral blood flow can be used to assess heart rate and to estimate HRV [23]. Several such devices conform with health, safety, and environmental protection standards. They can be adjusted by the user to different breathing rates but are usually set at a default rate of 6 breaths per minute. Assessment of the accuracy of some devices has also been carried out [48,49]. A growing number of research studies have also now investigated their effectiveness when used as stand-alone interventions.

There are wide variations in study design, using different devices, training protocols, and outcome measurements. Nevertheless, systematic reviews and meta-analyses have concluded that HRV biofeedback can be an effective treatment for symptoms in a range of different populations, including both adults [20,21] and children [50]. Providing interventions for symptoms such as anxiety that affect people with ASD has been emphasized as a vital area for research [51], and reviews have acknowledged the need to involve the ASD community directly in research [52,53]. HRV biofeedback has been used in a range of populations for anxiety management. To date, no studies have investigated the feasibility of using portable biofeedback devices as a home-based intervention to help people with ASD manage anxiety.

For people with ASD, biofeedback may provide specific advantages for the management of anxiety. First, it provides a technique for developing control over specific symptoms without the need for verbally based techniques designed for non-ASD populations or behavioral interventions that may be anxiety provoking for people with ASD [54]. Second, biofeedback can present structured visual information in a systematic manner, a factor suited to the typical communication strengths of people with ASD. Third, biofeedback also aims to increase awareness of physiological reactions, which can be reduced or impaired in people with ASD [55].

Finally, people with ASD show a range of different physiological reactions compared with non-ASD peers [56]. The different HRV responses of people with ASD have been debated [57,58]. Further investigation into interventions to help improve *interoceptive ability* or to develop physiological reactions may be particularly important for this population.

Accordingly, we proceeded with an exploratory study to investigate the use of HRV biofeedback outside of clinical settings as a potential intervention to help people with ASD by reducing symptoms of anxiety.

## Methods

### Overview

The aim of this pilot and feasibility trial comprised two main objectives: first, to explore the use of HRV biofeedback as a suitable methodology to support people with ASD to manage anxiety outside of clinical settings and, second, to assess the risks, benefits, and challenges of using HRV biofeedback within this population.

We involved adults with ASD and professionals working in the field in the initial study development and design. We then recruited a separate group of young people with ASD in an experimental design with appropriate pre- and postintervention outcome measures.

We recorded demographic data and mental health status and used participant anxiety and depression as the primary outcome measures. Statistical analyses were carried out to assess mean group differences in reported anxiety and depression. A per-protocol analysis was used. This led to several participants being excluded after randomization because they met the exclusion criteria, and their pre-post data were therefore not included in the quantitative data analysis. The pre-post data sets were analyzed using a standard statistical package (SPSS software [version 24.0; IBM Corp]). Correlational analyses were also used to review associations between baseline measurements and HRV data.

To assess the risks, benefits, and challenges we used several methods of data collection, including daily monitoring of device use, perceived participant stress levels using a questionnaire delivered through smartphone, standardized interviews, and short debriefing reports. Participants who dropped out early or had continuing difficulties using their biofeedback device or had electrocardiogram (ECG) recording difficulties after randomization had their monitoring data included for further analysis, provided that they had consented for these data to be collected. The aim of the study was to ensure methodological robustness and feasibility, with a focus upon potential risks, problems, or difficulties as well as potential benefits of using HRV biofeedback.

### Participants

The sample was drawn from a population of patients with an existing diagnosis of ASD who had attended regional health services for help with anxiety. The young people with ASD were invited to participate in experimental adoption of a portable HRV biofeedback device over a 12-week period. All participants in this study had been diagnosed with ASD in specialist health service assessments clinics using standardized measures. None had a learning disability, and all had attended mainstream education. Additional preassessment screening of participants was carried out using the *Social Communication Questionnaire* [59], yielding a mean score of 20, which is above the cutoff level for diagnosis of ASD. Participants were excluded if they had pre-existing addiction; a diagnosed cardiac condition; a learning disability; or where suicidal risk, psychosis, or severe eczema or psoriasis had been noted; or if they were taking medications known to suppress HRV, such as benzodiazepines or tricyclic antidepressants [60]. All participants received appropriate information about the study and gave consent or assent in addition to consent from a parent or carer. No incentives were offered for participation.

### Ethics Approval

Ethics approval was granted by the regional National Health Services ethics committee (15/NI/0255; IRAS: 139122). After review by the regional National Health Services ethics committee, approval was granted under the UK governance arrangements for research ethics committees.

### Recruitment

**Figure 1:**
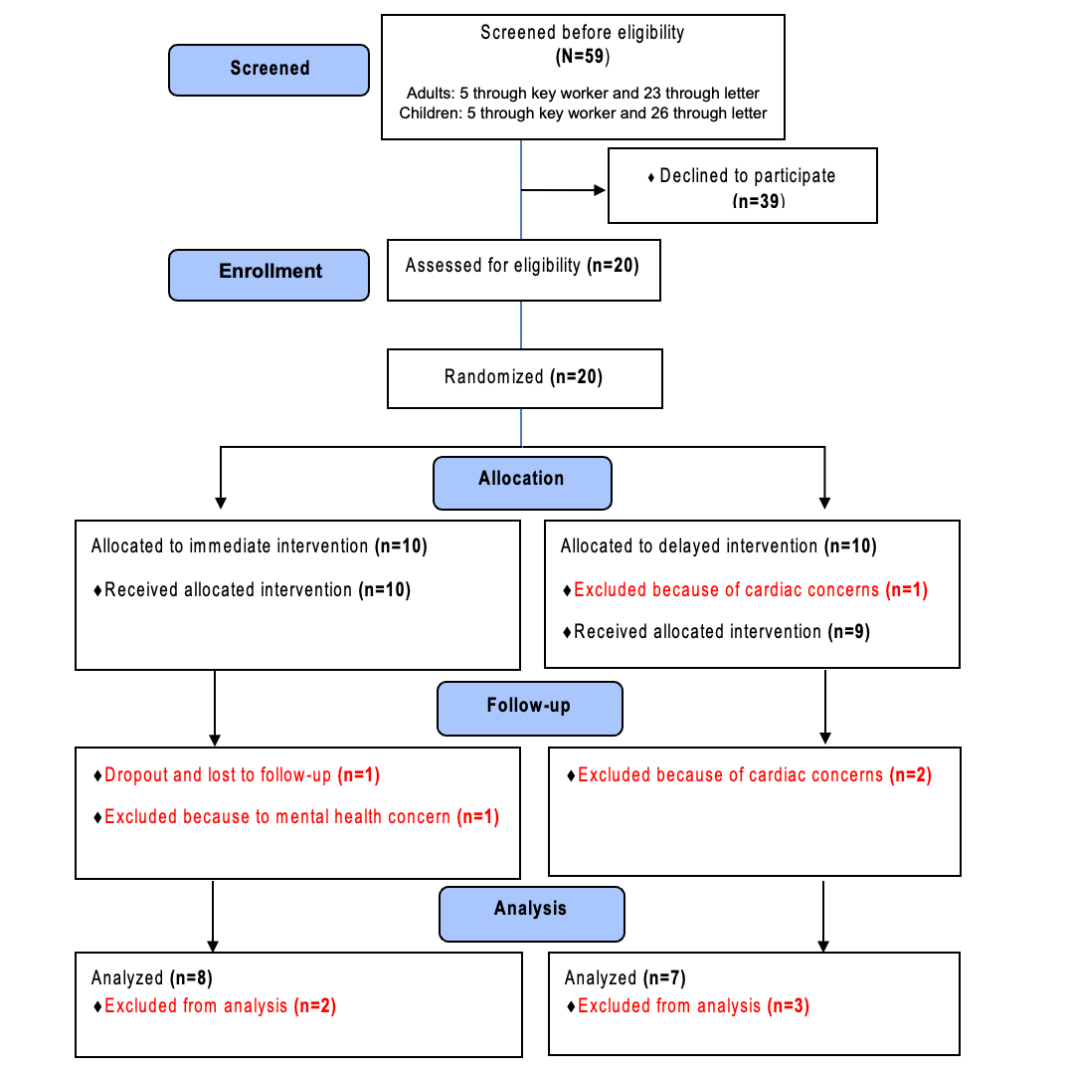
CONSORT (Consolidated Standards of Reporting Trials) flow diagram summarizing the processes adopted for screening and enrollment, allocation into groups, follow-up, and final analysis. Participant numbers are provided for each stage.

Potential participants were recruited using two methods: either through direct contact with their therapist or through letters sent to patients who were already discharged. It was not possible to determine the exact reasons for nonparticipation in the study because of the ethical constraints regarding contacting those who declined to participate through their therapist or those who did not respond to the invitation letters. Initially, 20 people took part: 16 (80%) male participants and 4 (20%) female participants. Their ages ranged from 13 to 22 (mean 16.2, SD 2.63) years. Detailed demographic information is presented in Multimedia Appendix 1 [61], and participant flow through the study is presented in Figure 1.

### Randomization

To decrease the risk of group allocation bias, random assignment to treatment group or control group was carried out *after* all preintervention assessments; therefore, the researcher was initially blind to who was allocated to each group during all baseline assessments.

Allocation was made in blocks to ensure that adequate numbers of participants were allocated to each condition. A randomized number sequence was generated through computer.

Between-group comparisons were planned to compare 6 weeks of intervention in the immediate group with 6 weeks of no intervention in the delayed group. The small sample size and additional exclusions from the study prevented these comparisons from being carried out.

### Equipment

**Figure 2:**
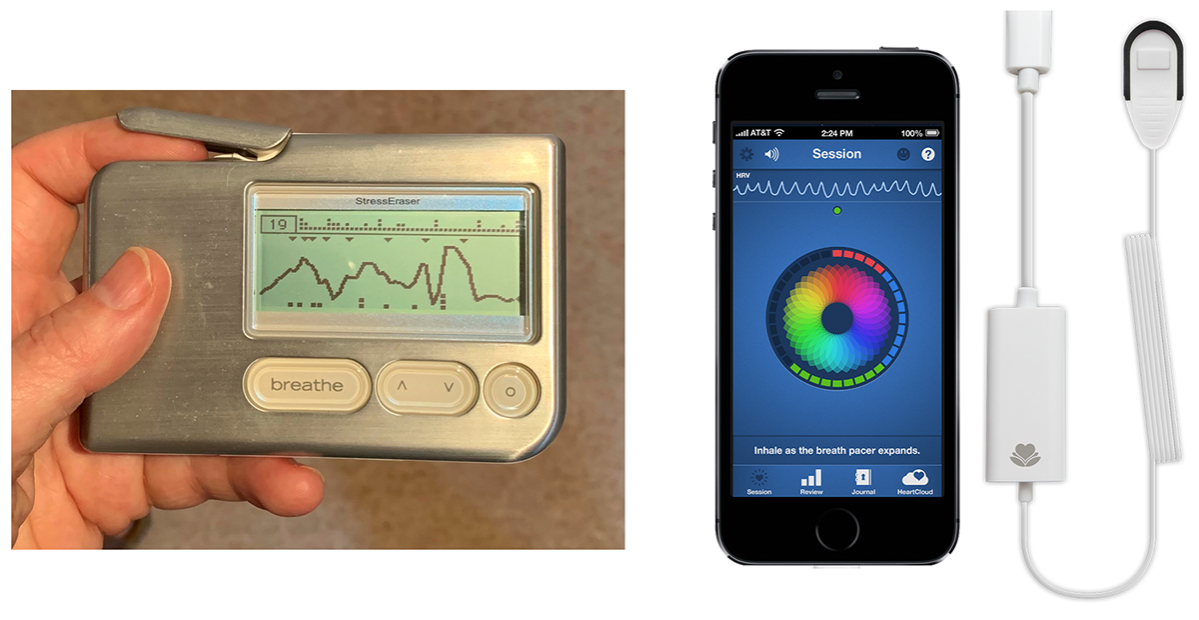
We used 2 personal home trainer devices to provide biofeedback during pilot testing: StressEraser, left, and Inner Balance, right (Inner Balance image reproduced with permission of HeartMath).

HRV home trainer devices differ in terms of form, feedback mechanisms, data storage, training guidelines, and underlying software, all of which may affect user experience and effectiveness. For this study, biofeedback devices were selected based on evidence of their use in previous research. To explore whether these aspects were of any concern, participants were allocated to 1 of 2 different biofeedback devices (Figure 2).

Group A participants were provided with a home trainer biofeedback device that used PPG ear sensors [62]. Group B participants were provided with a home trainer biofeedback device that used a fingertip PPG sensor contained within a stand-alone device [63]. A wireless single-lead ECG recorder, worn on the chest, was used to measure participant heart rate and HRV from a single lead before and after the intervention [64]. This lightweight device is battery powered and does not require any external leads or Velcro to connect to the recorder.

### Measures

Participants were invited to complete anxiety and depression questionnaires before and after the intervention. The measures included *Beck Anxiety Inventory* [65], *Beck Depression Inventory-II* [66], and *Beck Youth Inventories-II* for people aged 13 to 17 years [67] as appropriate to age. The parent or family carer was also invited to complete short before-and-after interviews, rating any changes in participant behavior over the course of the intervention period.

During the intervention period, participants were asked to complete short daily reports on stress levels and device use. This enabled us to track participant stress levels and monitor perceived usefulness of the device. The progress report was devised based on initial phase 1 evaluations and served a 2-fold purpose: tracking participant stress levels and monitoring use of the device over the course of the intervention. This short report asked questions on sources of stress, levels of stress, and use of the biofeedback device.

At the end of the intervention, participants and carers independently completed a short debriefing interview, and participants completed equipment usability ratings using the *System Usability Scale* [SUS]. This questionnaire provides a standardized assessment of the usability of different technology products [68]. The scale comprises 10 short statements rated on a 5-point scale. Item scores are summated and then rescaled in the range from 0 to 100, with higher scores indicating higher usability. Empirical evaluations of the SUS indicate good reliability [69]. A summary of the measures used is provided in Multimedia Appendix 2 [70,71,65,66,67,72,73,68].

### Intervention

All participants were offered initial assessment and training in their own home, with their carer present. This was carried out by a chartered clinical psychologist with certified training in HRV biofeedback (HC). The training involved sending video clips and written instructions produced by the device manufacturers to participants before an agreed home visit in which a direct demonstration of device use was given. Participants were then all seen and assessed by the first author (HC) who offered 2 training sessions (lasting for 30 minutes each) of home instruction in use of the allocated biofeedback device. No other intervention regarding anxiety management was provided. The HRV biofeedback intervention was available to use daily for a period of 12 weeks. Self-tailoring of intervention intensity was allowed to gauge acceptability and record self-reported compliance. Use was encouraged by the first author (HC) at the outset and indirectly through monitoring uptake. Information on participant anxiety and depression was collected through questionnaire measures completed before and after the intervention.

Participant HRV was measured in the home through a psychophysiological stress profile assessment using a single-lead ECG recorder before and after the intervention. Once the intervention commenced, remote monitoring continued daily, using SMS text message prompting at preset intervals agreed with participants. Questions used to monitor use of the intervention were sent to participants at an agreed time in the evening, recording whether they had used the device that day and whether it had been perceived as useful. Finally, additional information on risks, perceived problems, and benefits of the intervention and equipment usability was collected in face-to-face interviews with participants and their carers at the end of the intervention. This information was also collected from those who dropped out and those whose pre-post data were excluded, provided they consented to provide these data. This was seen as crucial to capture information on any potential difficulties and ensure a representative assessment of risks and benefits. Further details on the intervention are presented in the CONSORT (Consolidated Standards of Reporting Trials) and Template for Intervention Description and Replication checklists (Multimedia Appendices 3 and 4) and the study protocol (Multimedia Appendix 5 [68,74,75,76,64,77,73,78,79,24,80,81,82]).

## Results

### Demographic Information

All participants were nonsmokers, and none reported taking illegal drugs. Of the 20 participants, 10 (50%) reported prescribed medication. Of these 10 participants, 6 (60%) were prescribed selective serotonin reuptake inhibitor antidepressants, and 4 (40%) took stimulant medication. Sleep disturbance was reported by 75% (15/20) of the participants; 15% (3/20) reported needing to carry auto injectors with adrenaline (EpiPen). Of the 20 participants, 16 (80%) were employed or were students.

Carers were asked standardized questions regarding their main concerns about participant behavior, including whether there were any triggers for participant anxiety attacks or *meltdowns* and any strategies used to manage anxiety. The main concern reported was anxiety. The main triggers for anxiety attacks reported by 70% (14/20) of the families were sensory issues such as “loud noise,” “bright lights,” or “touch”; other triggers reported were “changes in routine” and increases in workload or examinations. Busy or crowded places, for example, “shopping centers” or *school*, were also mentioned as frequent triggers for anxiety by 55% (11/20) of the families. The most frequently reported strategy to help manage anxiety described by 85% (17/20) of the families involved reducing sensory information; for example, “reduce noise” or “turn down lights.” Other strategies reported were engaging in favorite activities or physical activity, spending time with parent or carer or pet, listening to music, and escaping from the source of stress by fighting or running away. All carers and participants reported more than one strategy for managing anxiety.

### Participant Anxiety and Depression

Of the 20 participants, 4 (20%) were excluded after randomization because of the identification of cardiac concerns (n=3, 75%) and significant mental health concerns (n=1, 25%), whereas 1 (5%) dropped out and declined further questionnaire and physiological assessment, leaving 15 participants (n=11, 73%, male participants and n=4, 27%, female participants) for before-and-after data analysis (Figure 1).

Statistical analyses were carried out using SPSS software (version 24.0) to assess mean group differences in reported anxiety and depression. Parametric statistical tests were conducted using paired sample 2-tailed *t* tests. Anxiety scores at baseline did not deviate from normality on either the Kolmogorov-Smirnov test (*P*=.20) or the Shapiro-Wilk test (*P*=.23). As different measures were necessarily used for children and adults, separate analyses were performed for these groups.

Of the 15 participants, 1 (7%) adult participant did not complete the initial depression questionnaires: 14 sets of depression data were analyzed. Parametric statistical tests were also conducted using paired sample 2-tailed *t* tests. Again scores at baseline did not deviate from normality on either the Kolmogorov-Smirnov test (*P*=.20) or the Shapiro-Wilk test (*P*=.78).

Data collected from children and adults showed statistically significant reductions in mean score for anxiety after the intervention; the results for both adults and children showed no significant reduction in mean scores for depression (Table 1).

**Table 1:** Participant questionnaire data showing mean scores for anxiety and depression before and after using heart rate variability biofeedback (N=15).

Participants (aged 13-24 years)		Before the intervention, mean (SD)	After the intervention, mean (SD)	Mean difference (SD)	Coefficient, *r*		*t* test (*df*)	*P* value (2-tailed)	Cohen *d* (adjusted)
**Anxiety**									
	BYI-A^a^ (children: n=7)	24.43 (8.98)	14.43 (10.97)	10.00 (10.39)		0.472	2.55 (6)	.04	0.99
	BAI^b^ (adults: n=8)	21.12 (11.2)	15.00 (11.49)	6.12 (4.39)		0.925	3.95 (7)	.006	0.54
**Depression**									
	BYI-D^c^ (children: n=7)	20.43 (14.04)	13.71 (14.16)	6.71 (9.53)	0.077		1.86 (6)	.11	0.48
	BDI^d^ (adults^e^: n=7)	17.0 (12.74)	13.86 (10.91)	3.14 (4.85)	0.928		1.72 (6)	.14	0.25

^a^BYI-A: Beck Youth Inventory, Anxiety scale.

^b^BAI: Beck Anxiety Inventory.

^c^BYI-D: Beck Youth Inventory, Depression scale.

^d^BDI: Beck Depression Inventory.

^e^An adult participant did not complete the depression questionnaire.

### Carer Reports on Behavior

Carers were asked to rate the frequency of participant behavioral outbursts or meltdowns at the initial and debriefing interviews. Carer ratings indicated a significant reduction (Wilcoxon signed-rank test) in the frequency of behavioral outbursts comparing initial interview data with debriefing interview data (*Z*_14_=–3.33; *P*=.001; *r*=0.6).

### ECG Assessment

Attempts were made to record wireless ECG data to detect changes in HRV over time using a psychophysiological stress test [24] before and after the intervention, but it was not possible to standardize this procedure because of the wide variations in the timing of assessments, differing home environments, and the use of HRV data from both children and adults. However, baseline ECG recordings did enable within-subject comparisons. Pearson bivariate correlations compared heart rate and HRV variables with age and level of ASD symptoms (Table 2). Before the intervention, age was significantly negatively correlated with all HRV variables at baseline, namely high-frequency heart rate variability (*P*=.02), the root mean square of successive differences in normal heartbeat contractions (*P*=.02) and the variability of normal-to-normal interbeat intervals (*P*=.04). ASD symptoms were positively correlated with heart rate both before (*r*=0.54; *P*=.04) and after the intervention (*r*=0.74; *P*=.001), and negatively correlated with HF HRV recorded at the final assessment (*r*=–0.56; *P*=.03). In addition, HRV indices were negatively correlated with heart rate both before and after the intervention.

**Table 2:** Pearson bivariate correlation statistics before and after the intervention between physiological measures of heart rate and heart rate variability recorded with participant age and level of ASD (autism spectrum disorder) symptoms as measured by the Social Communication Questionnaire [59].

			Age		ASD		HF-HRV^a^		RMSSD^b^	SDNN^c^		HR^d^
**Age**												
	Before the intervention	1		–0.250		–0.580^e^		–0.596^e^		–0.548^e^	–0.001	
	After the intervention	—^f^		—		–0.389		–0.381		–0.522^e^	–0.062	
**ASD^g^**												
	Before the intervention	—		1		–0.221		–0.134		–0.184	0.541^e^	
	After the intervention	—		—		–0.557^e^		–0.442		–0.504^e^	0.743^h^	
**HF-HRV**												
	Before the intervention	—		—		1		0.929^h^		0.902^h^	–0.562^e^	
	After the intervention	—		—		—		0.892^h^		0.905^h^	–0.733^h^	
**RMSSD**												
	Before the intervention	—		—		—		1		0.946^h^	–0.514^e^	
	After the intervention	—		—		—		—		0.910^h^	–0.616^e^	
**SDNN**												
	Before the intervention	—		—		—		—		1	–0.465	
	After the intervention	—		—		—		—		—	–0.694^h^	
**HR**												
	Before the intervention	—		—		—		—		—	1	
	After the intervention	—		—		—		—		—	—	

^a^HF-HRV: high-frequency heart rate variability (a frequency domain index that can indicate parasympathetic nervous system activity).

^b^RMSSD: root mean square of successive differences in normal heartbeat contractions or interbeat intervals measured in milliseconds (a time domain heart rate variability index that can be associated with parasympathetic nervous system activity).

^c^SDNN: SD of the normal heartbeat contractions; that is, normal-to-normal interbeat intervals measured in milliseconds.

^d^HR: heart rate measured in beats per minute.

^e^*P*<.05 (2-tailed).

^f^Not applicable.

^g^Autism spectrum disorder symptomatology measured using the Social Communication Questionnaire [59].

^h^*P*<.01 (2-tailed).

### Adoption of Biofeedback Device During Intervention

Across the study, 474 web-based surveys were collected providing data on sources of stress and use of biofeedback devices throughout the intervention period. Participants who had difficulties using their biofeedback device or ECG recorder had their monitoring data included, provided they had consented.

Participants were asked during the intervention period whether the device had helped them when used. The majority (311/474, 65.6%) of the reports provided indicated that the biofeedback device had been used and that “it helped” when used. Only 4% (19/474) of the reports indicated that the device “didn’t help” when used. Regarding use, 88% (417/474) of the reports indicated that the device had been used for between 0 and 10 minutes, with 12% (57/474) of the reports indicating that the device had been used for >10 minutes.

Participants were also asked to report the reasons for not using the device. A summary of 144 reports provided by participants detailing a range of reasons is presented in Table 3.

**Table 3:** Participant reported effect of biofeedback device when used (n=144).

Reported reason for not using device	Responses, n (%)
“Not stressed” today	65 (45.1)
No clear reason given	24 (16.7)
“Busy” or “away”	20 (13.9)
“Device not with me”	17 (11.8)
“Forgot to use”	9 (6.2)
“Practicing later”	6 (4.2)
“Error on device”	3 (2.1)

The most frequent reason reported for not using the device was simply that the participant did not feel stressed. Other reasons reported were “too busy” (20/144, 13.9%), “device not with me” (17/144, 11.8%), “forgot to use it” (9/144, 6.3%), “practicing the device later” (6/144, 4.2%), and “error on device” (3/144, 2.1%). Of all reports submitted, 16.7% (24/144) offered no clear reason for not using the device.

### Equipment Usability

Usability ratings were captured on the 2 biofeedback devices, an ECG recorder, and a web-based survey (Table 4). The benchmark for the SUS is 68, with scores above this value considered to be acceptable [83]. Average ratings for the equipment used in this study indicated good usability.

**Table 4:** Participant ratings of 2 types of biofeedback devices, an electrocardiogram (ECG) recorder, and SMS text monitoring using the System Usability Scale (SUS) [68].

Type of equipment used^a^	SUS score, mean (SD; range)	Benchmark SUS score^b^, mean (SD)
StressEraser (n=9)	76.60 (16.25; 47.5-92.5)	68 (12.5)
Inner Balance (n=9)	83.61 (8.94; 70.0-92.5)	68 (12.5)
Actiwave ECG recorder (n=9)	70.00 (7.80; 60.0-77.5)	68 (12.5)
SMS text message survey (n=15)	78.50 (9.05; 65.0-100.0)	68 (12.5)

^a^A total of 18 ratings of biofeedback devices were completed: 9 for StressEraser and 9 for Inner Balance; 9 ratings were completed regarding the electrocardiogram recorder; and 15 ratings were completed regarding the SMS text message report.

^b^Benchmark calculation of average SUS scores [83].

### Risks and Benefits

During the debriefing at the end of the study, participants and carers reported perceived benefits and any problems. Unexpectedly, many of the participants reported cold fingers during initial training, with 70% (7/10) of those initially allocated to the StressEraser reporting difficulty using this device at some point during the study.

Clinical disclosures included previously unrecognized cardiac irregularities (3/20, 15%) and severe mental health difficulties (1/20, 5%). These were identified *after enrollment* after initial screening.

A range of positive benefits were reported by both participants and their carers, with the most frequent benefit reported being feeling *calm* or *relaxed*. A smaller number of problems were reported overall, mainly relating to difficulties with the fingertip sensor device. The results presented in Tables 5 and 6 show the frequency counts of key phrases reported in debriefing reports from participants.

**Table 5:** Participant reported problems of using biofeedback device (n=24).

Reported problems	Responses, n (%)
Finger sensor errors	5 (21)
Difficulty using while stressed	5 (21)
Lack of practice	4 (16)
Device functions difficult	3 (13)
Needed reminders	3 (13)
Didn’t find it helped	2 (8)
Ear sensor difficulty	2 (8)

**Table 6:** Participant reported benefits of using biofeedback device (n=43).

Reported benefits	Responses, n (%)
Felt calm or helped	11 (25)
Ease of use	9 (21)
Helped breathing	5 (11)
Helped sleep	4 (9)
Good video tutorials provided	3 (7)
Visual or can “see” results	3 (7)
Helped when not using	2 (5)
Helped focus	2 (5)
Portable	2 (5)
Efficient	2 (5)

## Discussion

### Principal Findings

This pilot study highlights potential positive effects of HRV biofeedback in people with ASD, with fewer symptoms of anxiety being reported after using HRV biofeedback devices at home. Carers also reported fewer behavioral *meltdowns*. Remote monitoring indicated that although most (417/474, 88%) of the participants only used the device for short periods of time (0-10 minutes), the majority (311/474, 65.6%) indicated that the device helped when it was used. Notably, only 4% (19/474) of the reports highlighted instances where the device did not help. However, difficulties surrounding the usability of the finger-type sensor device were reported.

A perceived strength of this study was the experimentation of a new intervention in a population where there is high need and where little research exists on issues that affect the lives of people with ASD [84]. A further strength was the assessment of people with ASD by embedding self-reporting in place of traditionally used carer reports. Indeed, self-reports from young people with ASD can be reliable over time [85], and the use of remote monitoring, self-report questionnaires, and debriefing reports from participants combined with carer reports adds strength to the validity of the study findings. Overall, the findings confirmed that *young* *people with ASD can independently use portable HRV biofeedback devices, and such devices seem to help them manage anxiety.*

We note the discovery of previously undetected cardiac irregularities and deteriorating mental health. This highlights the mental and physical health vulnerabilities in this population [86,87] and the importance of providing them with both assessment and ongoing support. Pre- and postintervention data also indicated that a child participant and an adult participant showed increases in anxiety after the intervention. The debriefing reports indicated that usability issues with the device may have increased anxiety. The unforeseen instances of participants presenting with cold fingers, which affected the quality of the signal acquisition using the PPG finger sensor integrated into the StressEraser, was an unexpected finding. Cold fingers in some individuals have previously been correlated with a shutdown in peripheral circulation as a result of stress [88]. Equipment checks did not indicate any fault with the equipment, and this device has previously been used successfully in several other populations [89,90]. These difficulties highlight the importance of personalizing digital health solutions to promote use and adoption alongside the need for ongoing clinical support when problems occur. Although the StressEraser is no longer being marketed, other devices using finger sensors may need to be used with caution in people with ASD.

### Limitations

Several limitations are acknowledged in this pilot study. A necessary limitation was the small sample size and the inherent risk of error and bias when using self-report measures. It was not possible to determine the exact reasons for nonparticipation in the study because of the ethical constraints regarding contacting those who declined to participate. All participants contacted had already attended services for anxiety. It may be that those who did not participate were not now experiencing problems with anxiety or that they were concerned about participating in research into an untested intervention. Future studies should address this issue to further assess whom this treatment might be beneficial for.

It is possible that reductions in self-reported anxiety may have been related to other outside factors or to nonspecific therapeutic variables. However, it is of note that no time was spent with participants talking about their anxiety or providing any other type of intervention—the training given only involved a review of existing instruction guidelines in the use of each biofeedback device. It is also possible that unconscious bias may have occurred within carer interviews and participant reports because of a wish for the treatment to succeed; however, the debriefing reports indicated that several participants and their carers did report at the end of the intervention that the device did not help despite their initial hopes that it would be beneficial, and participant reports and carer reports were in concordance regarding the changes noted.

This study attempted to carry out before-and-after HRV assessment analysis by means of a single-lead ECG recorder using a psychophysiological stress test within participants’ homes. This type of test paradigm assessment proved unsuccessful because of the lack of standardized test conditions inherent within multiple home environments. This type of stress assessment is one which is unlikely to be useful outside of clinical settings. Before embarking on home intervention programs, future interventions using physiological monitoring such as ECG assessments may require an initial clinic appointment to carry out full mental and physical health checks [75].

In addition, individuals with ASD have also been found to exhibit both hyperarousal and hypoarousal responses to stress tasks, suggesting that the classic paradigm of a stress profile assessment designed for non-ASD populations is unlikely to provide a clear picture when used in people with ASD [56,91]. Recent research has suggested that it is preferable to carry out a longer assessment to obtain more accurate information on HRV, particularly in relation to psychological stress [92]. Because of the mental health difficulties and physiological differences observed in many people with ASD, this type of longer-term monitoring could be particularly valuable.

Usability assessment indicated that both ECG monitoring and remote smartphone monitoring of stress levels were found to be acceptable in people with ASD. Small wireless ECG monitors can now be used for 24-hour recordings, and the approach of remote stress monitoring combined with longer ECG recordings could be used in future studies to provide much needed data on anxiety and the physiological profile of people with ASD.

### Comparison With Prior Work

A review of HRV biofeedback studies [93] concluded that, although positive outcomes are reported in many studies, there is not always concordance between questionnaire reports and physiological measurements, and the exact mechanisms of the effect underlying HRV biofeedback have been debated [46]. Our findings suggest that there may be anxiety reduction through use of smaller portable HRV biofeedback devices, but further research using standardized physiological assessments is needed to establish whether this has any long-term effect on underlying participant HRV.

Importantly, some participants in this pilot may not have been able to develop the specific type of resonant frequency breathing using home trainer biofeedback devices, which is argued to increase HRV [94]. The use of multichannel biofeedback equipment may be important for initial training sessions to increase HRV levels; however, for the large numbers of people now requiring intervention, this would require significant outlays in terms of time, training, and resources. Future studies could carry out initial psychophysiological monitoring in a controlled clinic setting and then provide participants with wearable technologies for home use.

This preliminary work has provided vital information for further studies, which could now test effectiveness of HRV biofeedback for home-based remote management of anxiety in an adequately powered randomized trial using a comparator intervention with matched intervention time. This approach was used with non-ASD populations in 2 recent studies that reported positive effects from biofeedback in comparison with control interventions, such as mindfulness and walking [90,95].

Studies investigating comparative interventions could use a breathing pacer app for smartphone use, which could assess paced breathing alone in comparison with HRV biofeedback. Future investigations should capture device data, enabling information to be gathered regarding length and type of breathing practice, which may help to address questions relating to any dose-response relationship for HRV biofeedback. Finally, this initial study only targeted young people aged 13 to 24 years with no known learning disability. A key step in further work will be to assess this methodology and intervention in other groups of people with ASD, such as older adults and people with intellectual disability.

### Conclusions

ASD is now a common condition. Reports suggest high costs of supporting people with ASD; yet, little research has been undertaken into new types of interventions specifically designed to meet their needs [51].

Conditions such as ASD pose a significant cost to individuals, health care providers, and society as a whole [96]. Since the data were collected, limited access to interventions for mental health conditions such as anxiety has been exacerbated by health service imperatives to address the COVID-19 pandemic. It has been argued that the current health crisis demands a “digital mental health revolution” [97]. Digital health can provide important tools to help reduce the burden of mental illness and intervene with increasing numbers of people whom traditional models of *face-to-face* therapy cannot support [98].

Digital technology may represent a useful method of engaging people with ASD by using some of their characteristic strengths and interests, without the complex social and communication demands of traditional cognitive and behavior therapies [54] and without the potential risks of medication.

The application of home-based solutions to difficulties experienced by people with ASD also represents what has been termed a “naturalistic developmental behavioral intervention” that can help with the generalization of skills because of their use in real-world interactions [99]. Methods of remote monitoring to assess the symptoms as well as the effectiveness of interventions such as biofeedback may be useful for people with ASD, which should be trialed. These techniques may also assist practitioners striving to provide personalized *connected health* ASD intervention support at a distance.

Systematic reviews have outlined developments in biofeedback across a range of modalities as well as some of the challenges to be addressed in future investigations [28]. HRV biofeedback may be an important adjunct to existing interventions for non-ASD populations with mental health conditions [100,101], and people with ASD should not be excluded from developments in this area. Despite the limitations, this study provides preliminary information on the use, risks, and perceived benefits of HRV biofeedback for anxiety in people with ASD. Importantly, the direct reports from both participants and their carers provide a unique insight into the risks and difficulties as well as the potential benefits of this intervention. Although the exact mechanism of the effect remains unclear, our findings suggest that further research is warranted to clarify its effectiveness.

## Appendix

Multimedia Appendix 1 Participant demographic information.

Multimedia Appendix 2 Measures.

Multimedia Appendix 3 CONSORT (Consolidated Standards of Reporting Trials) 2010 checklist of information to include when reporting a randomized trial.

Multimedia Appendix 4 The Template for Intervention Description and Replication checklist.

Multimedia Appendix 5 Study protocol.

## Abbreviations

ASD: autism spectrum disorder
CONSORT: Consolidated Standards of Reporting Trials
ECG: electrocardiogram
HRV: heart rate variability
PPG: photoplethysmography
SUS: System Usability Scale
