# Supplementary material for: Heart Rate Variability Biofeedback to Treat Anxiety in Young People With Autism Spectrum Disorder: Findings From a Home-Based Pilot Study
Source: JMIR Form Res. 2022 Aug 26;6(8):e37994. doi: 10.2196/37994 (PMC9463620; doi:10.2196/37994)
Supplement: Multimedia Appendix 1 [file formative_v6i8e37994_app1.docx]

**Appendix 1 - Participant Demographic information**

|  | Number | (Percentage) |
| --- | --- | --- |
| *Gender* |  |  |
| Male | 16 | (80) |
| Female | 4 | (20) |
| *Age when diagnosed with ASD* |  |  |
| Under 5 years | 1 | (5) |
| 5-11 years | 13 | (65) |
| 12-17 years | 6 | (30) |
|  |  |  |

| *Type of education or employment* |  |  |
| --- | --- | --- |
| Not in education or employment | 4 | (20) |
| Secondary / grammar school | 13 | (65) |
| College / university | 3 | (15) |
|  |  |  |

| *Levels of physical activity* |  | | |  |
| --- | --- | --- | --- | --- |
| Less than 1 hour per week | 8 | | | (40) |
| 1-2 hours per week | 6 | | | (30) |
| 2-3 hours per week | 4 | | | (20) |
| 3+ hours per week | 2 | | | (10) |
|  |  | |  | |
| *Level of sleep problems* |  | |  | |
| No problem sleeping | 5 | | (25) | |
| Sometimes problems sleeping | 5 | | (25) | |
| Frequent problems sleeping | 10 | | (50) | |
|  |  |  | | |
| *Prescribed medication^1^ taken by participants.* |  |  | | |
| Not taking medication | 10 | (50) | | |
| Antidepressant | 6 | (30) | | |
| Stimulant medication | 4 | (20) | | |
| EpiPen | 3 | (15) | | |
| Melatonin | 3 | (15) | | |

^1^Several participants were taking more than one form of prescribed medication. None of the participants reported smoking or taking drugs. Two participants in the adult group reported drinking alcohol at a level which was within the UK Department of Health (DOH) recommended limit of 14 units per week [61].
