# Supplementary material for: Heart Rate Variability Biofeedback to Treat Anxiety in Young People With Autism Spectrum Disorder: Findings From a Home-Based Pilot Study
Source: JMIR Form Res. 2022 Aug 26;6(8):e37994. doi: 10.2196/37994 (PMC9463620; doi:10.2196/37994)
Supplement: Multimedia Appendix 2 [file formative_v6i8e37994_app2.docx]

**Appendix 2 - Measures**

A summary of questionnaires and interviews used; person reporting, and time taken for each measure

| Stage of Intervention | Person  Reporting | Measure Used | Time needed^1^ |
| --- | --- | --- | --- |
| Pre- intervention* Baseline | Carer | *Demographic information and Carer Interview; Social Communication questionnaire (Rutter, M.D., 2003)*  *Adolescent/Adult Sensory Profile [71]* | 45 minutes  15 minutes |
|  | Participant | *Beck Anxiety Inventory [65]; [66] OR (Beck Inventory 13-17 years [67] Nijmegen Q’aire [72].* | 10 minutes  5 minutes |
|  |  | *HRV recording using single lead ECG [73]* | 15 minutes |
|  |  |  |  |
| Intervention  monitoring | Participant | *Online progress report via SMS text: monitoring of stress levels and use of device (monitoring report devised by first author listed below)* | 1 minute |
|  |  |  |  |
| Post intervention* | Carer | *Carer Interview*  *Debriefing interview* | 15 minutes 5 minutes |
|  | Participant | *Beck Anxiety Inventory [65]; [66] OR (Beck Inventory 13-17 years [67]* | 10 minutes |
|  |  | *Debriefing interview*  *System Usability Scale [68]* | 10 minutes  5 minutes |
|  |  | *HRV recording using single lead ECG [73]* | 15 minutes |

^1^A total time of 2 hours was allocated to each home visit to allow for all assessments and interviews to take place.

## Intervention monitoring via SMS text report: monitoring of stress levels and use of device

**How are your stress levels today?**

- **no stress**
- **slight stress**
- **moderate stress**
- **very stressed**
- **totally stressed**

**If you are stressed do you know what is making you stressed?**

- **School problem**
- **Work problem**
- **Home problem**
- **Relationship problem**
- **Other reason I am stressed _________________________**
- **Don’t know why I am stressed**

**Did you use your biofeedback device today?**

- **Didn’t use it**

**I didn’t use it because _____________________________**

- **I Used it and it helped**
- **I Used it and didn’t help**

**How long did you use your device for?**

- **Less than 5minutes**
- **5-10 minutes**
- **More than 10 minutes**
