# Supplementary material for: Heart Rate Variability Biofeedback to Treat Anxiety in Young People With Autism Spectrum Disorder: Findings From a Home-Based Pilot Study
Source: JMIR Form Res. 2022 Aug 26;6(8):e37994. doi: 10.2196/37994 (PMC9463620; doi:10.2196/37994)
Supplement: Multimedia Appendix 5 [file formative_v6i8e37994_app5.docx]

**Appendix 5 -** **Study protocol**

Provisional ethical approval from the Office for Research Ethics Committees Northern Ireland was granted in December 2015. Further clarification of participant information booklets and equipment was requested, and final approval was granted in January 2016 for commencement of Phase 2 of the study on 21^st^ March 2016.

*Initial assessment*

All assessment and training was carried out by the researcher. All participants were offered assessment and training in their home environment, with their carer present.

After further checks on exclusion criteria were made, consent and assent were obtained. All participants completed baseline questionnaires followed by a physiological assessment. Carers completed a standardised interview and questionnaires on social, communication and sensory difficulties.

*Training*

Each training session lasted for 30 minutes once per week for two weeks. Each session involved initial demonstration of the allocated device by the researcher and a review of device functions using existing training protocols, guidelines and video clips. This demonstration was then followed by an initial practice session involving the participant using the device with direct coaching from the researcher. Finally, a practice plan was agreed outlining a time for practice that suited the participants’ routine with a recommendation of at least 10 minutes’ practice per day.

*Intervention and Monitoring*

Following baseline assessment, participants were randomised into an immediate intervention group or a delayed intervention control group. Immediate intervention participants were then offered two separate 30-minute training sessions of home instruction in use of their allocated biofeedback device (either device A or B). Delayed intervention participants waited six weeks before being reassessed again on baseline measures and allocated a biofeedback device and provided with home training in its use. Once the intervention had commenced, all participants were asked to complete regular reports on their stress levels and how frequently they used the device.

*Debriefing*

All participants were reassessed after they finished the intervention by repeating the questionnaires and physiological assessment procedure. Participants then completed a short debriefing interview and a usability rating of their device [68]. Carers were asked the same standardised interview questions which had been completed at baseline and also completed a short debriefing interview. In order to control for differing home environments, the procedure followed a standardised format using the same initial interview script; assessment procedure; training guidelines and the same debriefing interview script.

*ECG Recording*

Measurement of heart rate and HRV in participants was carried out with adherence to guidelines for assessment and reporting of HRV [74] and guidelines on the reporting of HRV in psychiatric populations [75].

Set up of equipment took place whilst participants completed questionnaires. All equipment was cleaned with 70% isopropyl alcohol prior to each assessment and again after each use. Set up involved programming the ECG recorder by inputting start time, date and recording ID – only the participant’s PIN, gender and task number were entered. The ECG recorder then recorded participant heart beats using two electrodes attached to the chest connected by a short lead. The recorder identified heart beats and R wave peaks within the ECG signal to enable calculation of R-R intervals. The sampling rate was set at 1024Hz with a 10-bit resolution, a recommended rate for this type of assessment 76. A 50Hz band pass filter was applied to remove contamination from background power line interference.

Once set up was complete, the recorder was attached. To avoid contamination or cross infection new electrodes were used for each individual assessment.

Checks were made to ensure the participants skin was not broken or red prior to each assessment. Each participant’s skin was first cleaned with simple soap and water wipes, prior to electrode placement. Skin was then lightly wiped using specialist exfoliation pads designed for ECG assessments to remove any existing dead skin, sweat and sebum. Alcohol wipes were not used as it was advised that these would be more likely to cause irritation [64]. Electrodes were then applied to the central sternum area of the chest, according to manufacturer’s instructions 64. The first electrode was placed directly onto the centre of the chest and the recorder was then attached onto it. The second electrode was attached to the left side so that the ECG recorder was positioned across the chest in approximately V1 – V5 positioning – see Figure 4.9.

Two different placement positions are recommended as suitable depending on the user’s anatomy and size.

*Figure 4.9 Illustration of two different electrode placement positions for HRV assessment recommended for single lead ECG Actiwave Cardio recorder (reproduced with permission of CamNtech Ltd).*

To minimise problems with diurnal variation participants were seen at the same time of day for each repeat assessment, and records were made of any medications taken. Participants were instructed to sit upright at a dining room or kitchen table, in a straight-backed chair and watch a series of images displayed on a computer (PowerPoint~~™~~ presentation) via a 15-inch laptop.

Adjustments were made to the laptop height to ensure participants viewed the screen at a comfortable height without having to look up or down. The screen was positioned within one metre of each participant.

Participants were encouraged to remain seated and upright throughout the three assessment stages. Two 30 second breaks were allowed between individual sections of the assessment task, to allow for movements and stretching. Instructions for the task were presented on the laptop screen. Participants were reassured that the task was not a test and that they should simply relax and enjoy watching the images.

Deliberately controlling breathing rates and talking have been argued to alter HRV [77]. Therefore, instructions were given to ‘*just relax*’ and to breathe normally to ensure participants did not try to actively adjust their breathing rates. The software used to analyse ECG recordings produced a derived estimate of respiration to enable checks that breathing rates were within the normal parameters used for analysis of HRV [74]. Separate respiration gauge belts were not attached to participants following concerns about straps and Velcro reported in the Phase 1 evaluations.

The assessment lasted for a total of 14 minutes. Researcher notes were also made of any difficulties with the assessment and responses to the task, including problems with electrode placement and any excess movement. At the end of the recording, the recorder was removed from the electrodes and placed on its docking station which was connected to the laptop. Participants were given hypoallergenic wipes to aid removal of electrodes at the end of the assessment. Records were made of any reactions or reports of discomfort during attachment or removal of electrodes.

Finally, the ECG reader software downloaded data from the recorder to the laptop via the dock and USB cable. All ECG recordings were initially downloaded using the proprietary software [64] and saved as European Data Format files. A timing manager program recorded the exact viewing times of each PowerPoint slide thus enabling each section of the task to be recorded accurately.

HRV data was extracted from each of the three sections of the assessment and analysed using specialist software (Kubios HRV Premium version. 3.0.2) [73].

Artefact identification and correction was conducted on all HRV recordings by using a ‘smoothness priors based detrending approach’ to analysis by setting the software to ‘smooth priors’ and also by using the ‘automatic’ artefact correction [73]. Using this type of standardised approach to removal of artefact with HRV analysis software has been advocated as an appropriate alternative to using a manual editing process [73, 79].

*Psychophysiological assessment task*

The task used for assessment of HRV followed a standard ‘psychophysiological stress profile’ paradigm used for assessing resting state HRV developed after consultation with experts in this area^^[[1]](#footnote-1)^^.

Participant HRV was assessed for a 14-minute period at the beginning and at the end of the intervention. Delayed intervention participants were also assessed at the initial assessment six weeks prior to starting the biofeedback intervention, whilst immediate intervention participants were assessed after six weeks of intervention.

Thus, three separate assessments were planned for each participant. These additional assessments were carried out to enable group comparisons to be made between immediate and delayed groups, comparing six weeks of intervention (in the immediate group) to six weeks of no intervention (in the delayed group).

The task involved three separate parts, each part lasting a minimum of three minutes, with additional time allocated for set up and short breaks. The format for tasks followed a commonly used protocol of ‘*baseline assessment*’ task followed by a ‘*mild stress*’ task, and finally a ‘*recovery*’ task [24].

The mild stress task used was the ‘*Reading the Mind in the Eyes Test’*. This assessment has been used to assess emotion recognition has been found to be a more difficult task for people with ASD [80, 81].

The baseline and recovery tasks involved using images from the ‘*International Affective Picture System*’ [82] a database of visual images which have normative ratings regarding their emotional content. Images used were selected rated as having positive and non-social content.

First, participants watched a series of images of scenes from nature that did not contain any people or animals *–* this initial part of the recording enabled a baseline to be established and allowed participants to get used to the recording.

Second, a series of images from the ‘*Reading the Mind in the Eyes Test*’ or RMET [80] was presented. This part of the assessment involved showing a series of images of eyes from human faces. Participants were asked to choose one of four words on the screen which best described the emotion shown on the face. Images used were all taken from the images in this test and varied in each assessment.

The third and final part of the assessment task, involved presenting a further series of images from the ‘*International Affective Picture System*’ or IAPS which were rated as having positive and non-social content [82]

1. ^1^Personal correspondence with Richard Gevirtz, June 2015 [↑](#footnote-ref-1)
